# Admission Blood Glucose Associated with In-Hospital Mortality in Critically III Non-Diabetic Patients with Heart Failure: A Retrospective Study

**DOI:** 10.31083/j.rcm2508275

**Published:** 2024-08-05

**Authors:** Yu Chen, YingZhi Wang, Fang Chen, CaiHua Chen, XinJiang Dong

**Affiliations:** ^1^Department of Cardiac Surgery, Taizhou Hospital of Zhejiang Province, Affiliated to Wenzhou Medical University, 317000 Linhai, Zhejiang, China; ^2^Department of Cardiology, Shanxi Cardiovascular Hospital, 030024 Taiyuan, Shanxi, China

**Keywords:** in-hospital mortality, admission blood glucose, heart failure, MIMIC-III, nonlinear relationship, U-shape

## Abstract

**Background:**

Heart failure (HF) is a primary public health issue associated with a high mortality rate. However, effective treatments still need to be developed. The optimal level of glycemic control in non-diabetic critically ill patients suffering from HF is uncertain. Therefore, this study examined the relationship between initial glucose levels and in-hospital mortality in critically ill non-diabetic patients with HF.

**Methods:**

A total of 1159 critically ill patients with HF were selected from the Medical Information Mart for Intensive Care-III (MIMIC-III) data resource and included in this study. The association between initial glucose levels and hospital mortality in seriously ill non-diabetic patients with HF was analyzed using smooth curve fittings and multivariable Cox regression. Stratified analyses were performed for age, gender, hypertension, atrial fibrillation, CHD with no MI (coronary heart disease with no myocardial infarction), renal failure, chronic obstructive pulmonary disease (COPD), estimated glomerular filtration rate (eGFR), and blood glucose concentrations.

**Results:**

The hospital mortality was identified as 14.9%. A multivariate Cox regression model, along with smooth curve fitting data, showed that the initial blood glucose demonstrated a U-shape relationship with hospitalized deaths in non-diabetic critically ill patients with HF. The turning point on the left side of the inflection point was HR 0.69, 95% CI 0.47–1.02, *p* = 0.068, and on the right side, HR 1.24, 95% CI 1.07–1.43, *p* = 0.003. Significant interactions existed for blood glucose concentrations (7–11 mmol/L) (*p*-value for interaction: 0.009). No other significant interactions were detected.

**Conclusions:**

This study demonstrated a U-shape correlation between initial blood glucose and hospital mortality in critically ill non-diabetic patients with HF. The optimal level of initial blood glucose for non-diabetic critically ill patients with HF was around 7 mmol/L.

## 1. Introduction

Heart failure (HF) remains a prevalent cardiovascular disorder, affecting approximately 40 million individuals globally [1]. Additionally, it is a leading cause of morbidity and mortality, which continues to increase, particularly within the aging population. Despite advancements in treatment and prevention strategies, optimizing clinical management for HF patients remains an area that needs further study. Several current investigations have underscored the association between elevated glucose levels and adverse outcomes in HF patients [2], and expert consensus recommends maintaining glucose levels between 6.1 and 7.8 mmol/L in critically ill non-diabetic patients. However, the optimal blood glucose target for critically ill patients remains under debate, with some studies indicating a higher mortality rate for patients admitted to intensive care units (ICUs), ranging from 4% to 7%, compared to those treated in general wards [3, 4, 5]. This disparity highlights the necessity for exploring the relationship between admission blood glucose levels and hospital mortality among critically ill non-diabetic patients with HF.

Hence, this retrospective study examines the correlation between admission blood glucose and in-hospital mortality in critically ill non-diabetic patients with HF. The findings of our investigation could offer insights into improving glycemic control in clinical practice and lay the groundwork for future research.

## 2. Methods

Medical Information Mart for Intensive Care-III (MIMIC-III) is a public, free, accessible critical care data resource containing 46,520 patients admitted to an ICU at the Beth Israel Deaconess Medical Center (BIDMC) in Boston, Massachusetts from 2001 to 2012 (https://mimic.physionet.org/) [6]. One author (YC) approved extracting the data (certification number 43426766). The Institutional Review Board of the BIDMC approved data research. This research complied with the standards for improving the Reporting of Observational Studies in Epidemiology [7] and was conducted in accordance with the Helsinki Declaration. The Institutional Review Board disregarded the requirement for informed written consent.

### 2.1 Study Patients

**Fig. 1.**
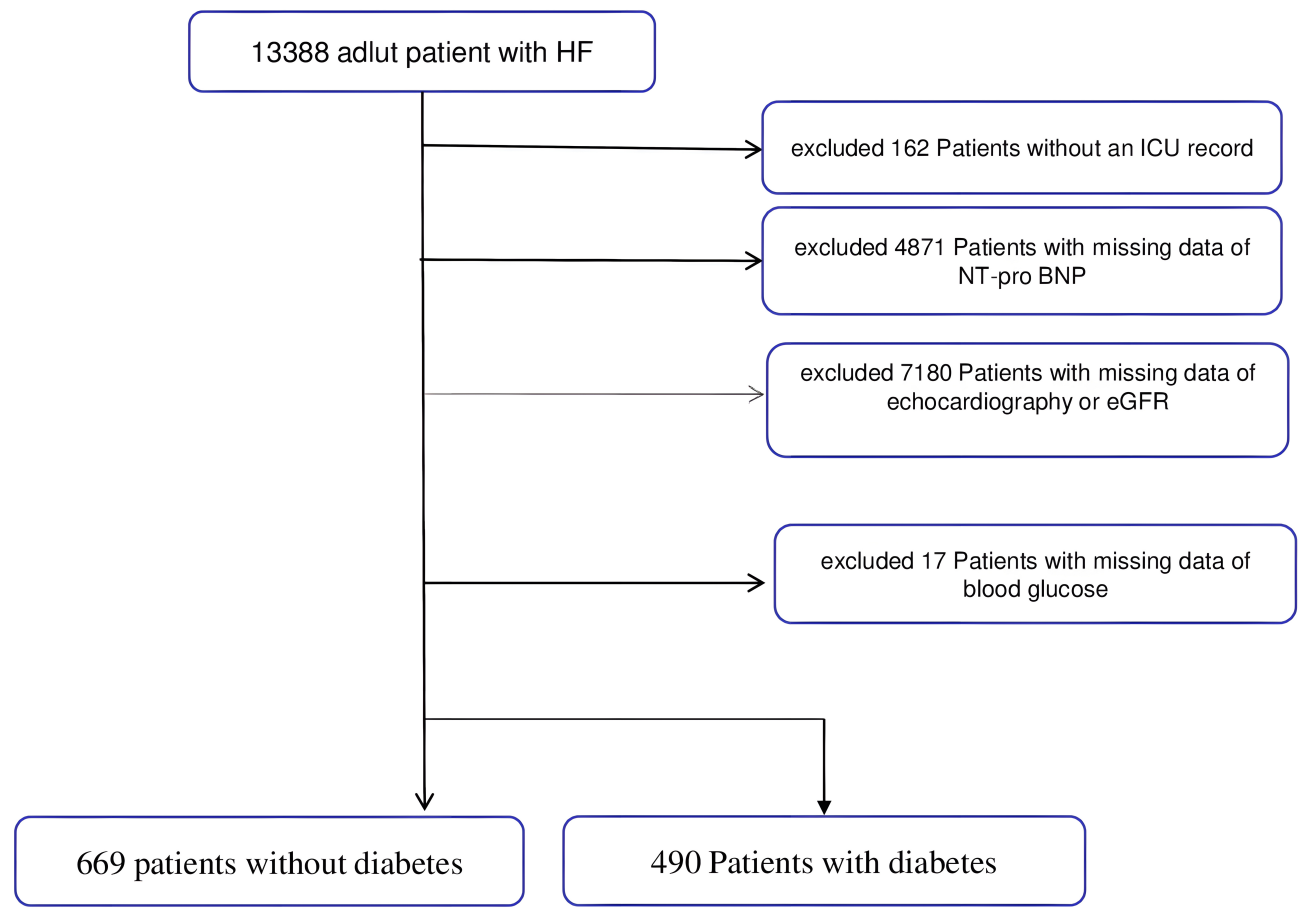
**Flow chart of patient disposition**. HF, heart failure; ICU, intensive care unit; eGFR, estimated glomerular filtration rate; NT-pro BNP, N-terminal pro b-type natriuretic peptide.

Subjects aged ≥18 years old, when admitted to the ICU and diagnosed with HF using the International Classification of Diseases, Ninth Revision (ICD-9) codes, were enrolled. Subsequently, 13,388 subjects diagnosed with HF were manually scanned, with 12,229 patients excluded for missing data (Fig. 1). The exclusion criteria included the following: No ICU record (n = 162), missing d NT-pro BNP values (n = 4871), missing echocardiography data or missing estimated glomerular filtration rate (eGFR) data (n = 7180), and those without an admission blood glucose reading (n = 17). Finally, 490 patients with and 669 without diabetes were analyzed (Fig. 1).

### 2.2 Data Extraction

Admission blood glucose: Following ICU admission, the venous blood was drawn to measure the initial blood glucose levels (admission glucose levels). We grouped admission blood glucose into quartiles. We categorized eGFR based on stages of chronic kidney disease (CKD): >90, 60 to 89, 45 to 59, 30 to 44, 15 to 29, <15 mL/min/1.73 $m^{2}$. Covariates: The following variables were included: Age, gender, body temperature, red blood cells (RBC), pulse oxygen (${\mathrm{SPO}}_{2}$), leucocytes, platelets, blood potassium, lactic acid, ejection fraction (EF), renal failure, cardiovascular heart disease (CHD) with no myocardial infarction (MI), chronic obstructive pulmonary disease (COPD), atrial fibrillation, hypertension, systolic blood pressure (SBP), diastolic blood pressure (DBP), and eGFR-outcome: in-hospital mortality.

### 2.3 Statistical Analysis

The findings were presented as SD ± mean or median. The correlation between initial glucose levels and in-hospital death was evaluated in critically ill non-diabetic patients suffering from HF using multivariable logistic regression models. Three models were constructed: A non-adjusted model, which unadjusted for any covariates; a minimally adjusted model, which adjusted for age and gender; a fully adjusted model, which fully adjusted for age, gender, EF, RBC, hypertensive, CHD with no MI, renal failure, COPD, atrial fibrillation, lactic acid, platelets, leucocytes, blood potassium, ${\mathrm{SPO}}_{2}$, temperature, SBP, and DBP. To account for the U-shape relationship between admission blood glucose and hospitalized mortality in critically ill patients with HF, a generalized additive model was performed along with the smooth curve fitting to address nonlinearity. A two-piecewise linear regression approach was adopted to assess the nonlinearity further. Subgroup analyses were performed to evaluate possible effect modifications using the covariables on the link between initial blood glucose and in-hospital death. The current study was conducted using R software, version 4.2 (http://www.r-project.org, The R Foundation) and Free Statistics software version 1.9 (FreeClinical Medical Technology Co, Ltd, Beijing, China), with *p* < 0.05 signifying statistical significance.

## 3. Results

### 3.1 Baseline Features of Participants

We identified 1159 patients with HF (Fig. 1). The average age of the subjects was 74.0 ± 13.5 years, and 47.5% were male. In the non-diabetic group, participants had a lower SBP; 14.9% of patients died while hospitalized. Table 1 lists the baseline features of the participants.

**Table 1.** **Initial features of the research participants**.

Covariates		Total (n = 1159)	Non-diabetic (n = 669)	Diabetic (n = 490)	*p*
Age (years)		74.0 ± 13.5	75.0 ± 13.9	72.6 ± 12.8	0.003
Gender n (%)					0.281
	Male	551 (47.5)	309 (46.2)	242 (49.4)	
	Female	608 (52.5)	360 (53.8)	248 (50.6)	
RBC (${\mathrm{10}}^{12}$/L)		3.6 ± 0.6	3.6 ± 0.6	3.5 ± 0.6	0.138
Leucocytes (${\mathrm{10}}^{9}$/L)		10.7 ± 5.2	11.0 ± 5.8	10.3 ± 4.4	0.017
Platelets (${\mathrm{10}}^{9}$/L)		241.7 ± 112.8	240.4 ± 116.1	243.5 ± 108.1	0.644
Blood potassium (mmol/L)		4.2 ± 0.4	4.1 ± 0.4	4.2 ± 0.4	<0.001
Ejection fraction (EF) (%)		48.7 ± 12.9	49.0 ± 12.8	48.3 ± 13.1	0.343
${\mathrm{SPO}}_{2}$ (%)		96.3 ± 2.3	96.1 ± 2.3	96.5 ± 2.3	0.022
Temperature (°C)		36.7 ± 0.6	36.7 ± 0.6	36.7 ± 0.6	0.405
SBP (mmol/L)		118.0 ± 17.4	116.0 ± 17.0	120.6 ± 17.6	<0.001
DBP (mmol/L)		59.6 ± 10.7	60.1 ± 10.7	58.9 ± 10.6	0.055
eGFR (mL/min/1.73 $m^{2}$)					0.022
	<15	93 (8.0)	41 (6.1)	52 (10.6)	
	15–29	239 (20.6)	138 (20.6)	101 (20.6)	
	30–44	260 (22.4)	153 (22.9)	107 (21.8)	
	45–59	191 (16.5)	126 (18.8)	65 (13.3)	
	60–89	196 (16.9)	112 (16.7)	84 (17.1)	
	>90	180 (15.5)	99 (14.8)	81 (16.5)	
Atrial fibrillation n (%)					0.673
	No	635 (54.8)	363 (54.3)	272 (55.5)	
	Yes	524 (45.2)	306 (45.7)	218 (44.5)	
CHD with no MI n (%)					0.539
	No	1064 (91.8)	617 (92.2)	447 (91.2)	
	Yes	95 (8.2)	52 (7.8)	43 (8.8)	
COPD n (%)					0.012
	No	1071 (92.4)	607 (90.7)	464 (94.7)	
	Yes	88 (7.6)	62 (9.3)	26 (5.3)	
In-hospital mortality n (%)					0.103
	No	1002 (86.5)	569 (85.1)	433 (88.4)	
	Yes	157 (13.5)	100 (14.9)	57 (11.6)	

All values are shown as mean ± SD, median or n (%). SBP, systolic blood pressure; DBP, diastolic blood pressure; COPD, chronic obstructive pulmonary disease; CHD, coronary heart disease; MI, myocardial infarction; RBC, red blood cells; ${\mathrm{SPO}}_{2}$, pulse oxygen; eGFR, estimated glomerular filtration rate.

### 3.2 Correlation between Initial Blood Glucose Levels and In-Hospital Mortality

The correlation between initial blood glucose levels and in-hospital mortality is shown in Table 2. A 1 mmol/L increase in admission blood glucose was 2% higher than in the associated in-hospital mortality: Adjusted for age, sex, EF, RBC, hypertension, CHD with no MI, renal failure, diabetes, COPD, atrial fibrillation, lactic acid, platelets, leucocytes, blood potassium, ${\mathrm{SPO}}_{2}$, temperature, SBP, and DBP—HR = 1.02; 95% CI, 0.9–1.16. In the sensitivity assessment, the admission blood glucose was also considered a categorical factor (quartile), and the *p*-value for this trend was 0.78.

**Table 2.** **The correlation between initial blood glucose and in-hospital mortality in non-diabetic critically ill patients**.

	Non-adjusted model	Minimally adjusted model	Fully adjusted model
	HR (95% CI)	HR (95% CI)	HR (95% CI)
Blood glucose	1.05 (0.95–1.16)	1.05 (0.95–1.16)	1.02 (0.9–1.16)
Q1	1 (Reference)	1 (Reference)	1 (Reference)
Q2	0.8 (0.44–1.44)	0.79 (0.44–1.44)	0.63 (0.3–1.34)
Q3	0.47 (0.24–0.91)	0.47 (0.24–0.92)	0.51 (0.23–1.11)
Q4	1.12 (0.64–1.95)	1.16 (0.66–2.03)	0.93 (0.46–1.88)

Non-adjusted model: no covariate was adjusted; minimally adjusted model: age and sex were adjusted; fully adjusted model: adjusted for age, gender, EF, RBC, hypertensive, CHD with no MI, renal failure, COPD, atrial fibrillation, lactic acid, platelets, leucocytes, blood potassium, ${\mathrm{SPO}}_{2}$, temperature, SBP, and DBP. SBP, systolic blood pressure; DBP, diastolic blood pressure; COPD, chronic obstructive pulmonary disease; CHD, coronary heart disease; MI, myocardial infarction; RBC, red blood cells; ${\mathrm{SPO}}_{2}$, pulse oxygen; HR, hazard ratio; CI, confidence interval; EF, ejection fraction.

### 3.3 The U-shape Association between Initial Blood Glucose Levels and In-Hospital Mortality

**Fig. 2.**
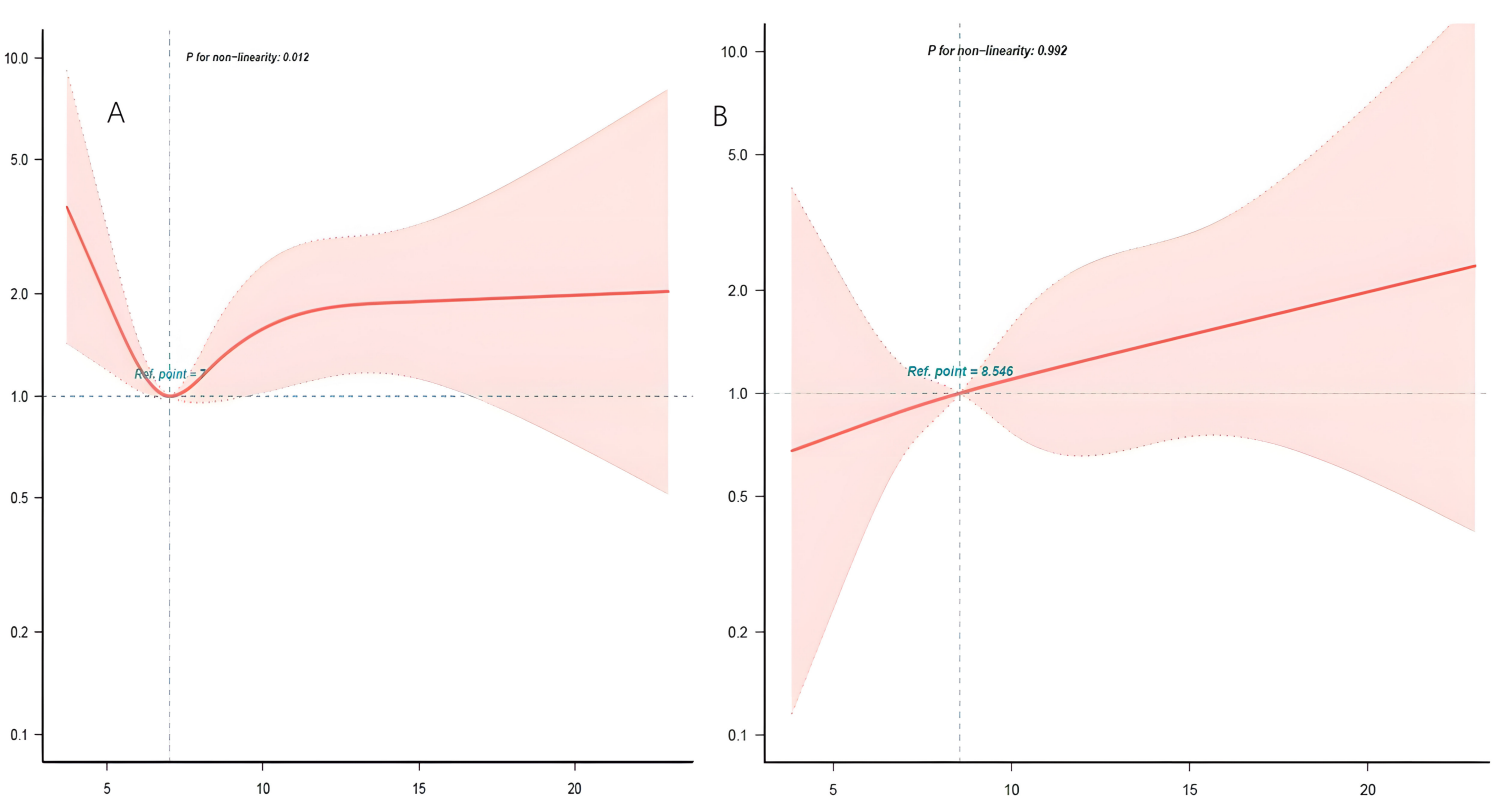
**The correlation between admission blood glucose and in-hospital mortality**. (A) The nonlinear correlation between the admission blood glucose levels and in-hospitalized mortality in non-diabetic patients. (B) The linear correlation between the admission blood glucose levels and in-hospitalized mortality in diabetic patients.

We found a U-shape association between initial blood glucose levels and hospital deaths using the multivariate Cox regression approach alongside a smooth curve fitting (Fig. 2). Through the two-piecewise linear regression model (Table 3), it was found that the inflection point was at approximately 7 mmol/L. The left inflection point was HR 0.69, 95% CI 0.47–1.02, *p* = 0.068, with the right: HR 1.24, 95% CI 1.07–1.43, *p* = 0.003. To examine the correlation between initial blood glucose and in-hospital death rate in various groups of non-diabetic HF patients, we performed exploratory subgroup analyses (Table 4). We used age, gender, blood glucose concentrations, eGFR, hypertension, atrial fibrillation, CHD with no MI, renal failure, and COPD as the categorization criteria for identifying the trend in the effect sizes within these parameters. Except for the blood glucose concentrations, there were no significant interactions in the above variables (*p* for interactions >0.05). When the patient’s blood glucose level is below 7 mmol/L (HR 0.7, 95% CI 0.47–1.03) or exceeds 7 mmol/L (HR 0.89, 95% CI 0.55–1.44), the association with in-hospital mortality appears to be non-significant. Conversely, a blood glucose range between 7 and 11 mmol/L demonstrates a positive correlation with in-hospital mortality: a 1% increase in blood glucose concentration is associated with a 55% increase in the risk of in-hospital mortality.

**Table 3.** **The nonlinearity correlation between the admission blood glucose level and hospitalized mortality**.

Thresholds of driving pressures		HR	95% CI	*p*-value
	<7	0.69	0.47–1.02	0.068
	≥7	1.24	1.07–1.43	0.003
Likelihood ratio test				0.007

HR, hazard ratio; CI, confidence interval.

**Table 4.** **Stratified analysis for the admission blood glucose and in-hospital mortality in non-diabetic heart failure patients**.

Subgroup		N (sample size)	OR (95% CI)	*p*-value	*p* for interaction
Age (years)					0.257
	<60	89	0.88 (0.64–1.22)	0.458	
	≥60, <70	111	0.86 (0.6–1.24)	0.424	
	≥70, <80	150	1.18 (0.99–1.4)	0.069	
	≥80	319	1.05 (0.91–1.21)	0.533	
Gender					0.719
	Male	309	1.07 (0.93–1.23)	0.334	
	Female	360	1.03 (0.89–1.19)	0.666	
Blood glucose concentrations (mmol/L)					0.009
	<7	336	0.7 (0.47–1.03)	0.068	
	7–11	290	1.55 (1.1–2.19)	0.012	
	>11	43	0.89 (0.55–1.44)	0.625	
eGFR (mL/min/1.73 $m^{2}$)					0.288
	<15	41	1 (0.7–1.42)	0.997	
	15–29	138	1.22 (0.96–1.53)	0.098	
	30–44	153	1.1 (0.93–1.29)	0.265	
	45–59	126	1.08 (0.86–1.36)	0.503	
	60–89	112	0.79 (0.55–1.14)	0.213	
	>90	99	0.84 (0.58–1.22)	0.373	
Hypertension					0.112
	No	222	0.95 (0.8–1.13)	0.539	
	Yes	447	1.12 (0.99–1.27)	0.063	
Atrial fibrillation					0.139
	No	363	1.12 (0.99–1.28)	0.083	
	Yes	306	0.96 (0.82–1.13)	0.625	
CHD with no MI					0.245
	No	617	1.07 (0.96–1.18)	0.221	
	Yes	52	0.83 (0.52–1.31)	0.425	
COPD					0.639
	No	607	1.06 (0.96–1.17)	0.281	
	Yes	62	1.18 (0.76–1.85)	0.461	

COPD, chronic obstructive pulmonary disease; CHD, coronary heart disease; MI, myocardial infarction; eGFR, estimated glomerular filtration rate; OR, odds ratio; CI, confidence interval.

## 4. Discussion

In this study, we utilized Restricted Cubic Spline (RCS) to identify the relationship between initial blood glucose and in-hospital mortality in critically ill non-diabetic patients with HF. There is a U-shaped relationship between initial blood glucose and hospital mortality in critically ill non-diabetic patients with HF. However, the initial blood glucose correlations with in-hospital deaths differed, with a turning point at about 7 mmol/L. The blood glucose levels at admission were not statistically significant on the left side of the inflection point but were positively correlated with the in-hospital death rate concerning the right side of the inflection point.

The correlation between blood glucose levels and in-hospital death in patients with congestive heart failure (CHF) is still unclear. The hospitalized mortality risk was elevated in patients with hypoglycemia [8, 9]. However, the definition of hypoglycemia among studies is inconsistent; most studies define hypoglycemia as a blood glucose level (<2.2 or <3.3–3.6 mmol/L). Additionally, hypoglycemia is currently defined by the World Health Organization (WHO) as a blood glucose level below 2.5 mmol/L and is associated with increased all-cause hospitalized mortality [10]. There are several plausible mechanisms: abnormal cardiac repolarization, sympathoadrenal activation, elevated inflammation, thrombogenesis, and vasoconstriction [11].

Several studies demonstrated that elevated blood glucose level is positively associated with the risk of HF in both women and men [12, 13]. Several mechanisms might explain this, including an inflammation reaction immunosuppression evoked by severe physiological and oxidative stress [14]. Hyperglycemia (HG) increases the risk of atrial fibrillation [15], hypertension [16], and coronary heart disease [17]. Excess circulating glucose results in lipid accumulation in the heart [18], collagen deposition and fibrosis, and insulin resistance. HG drives inflammation and oxidative stress, leading to endothelial dysfunction and increased cognitive impairment [19]. It is modified by hyperlactatemia and diabetic status, and the increased endothelial dysfunction plays an important role in the development of frailty [20]. HG is also recognized as a contributing factor to restenosis, causing harm to blood vessels and inducing microvascular lesions. Additionally, it is associated with heightened oxidative stress, enhanced inflammatory responses, and augmented platelet clumping [21]. Sodium-glucose cotransporter-2 inhibitor (SGLT2-I) gained intense interest in the search for the mechanisms responsible for their beneficial effects in patients with and without diabetes mellitus (DM) [22], although the precise mechanisms remain unclear.

A network meta-analysis was performed, which indicated that there was no mortality advantage associated with tight glycemic control in critically ill patients and was often tempered by concerns of inducing hypoglycemia [23, 24]. Administering empagliflozin effectively restored the diminished Sirt3 levels and corrected the abnormal glycolytic pathway. Moreover, this medication inhibited the build-up of glycolytic byproducts in diabetic kidneys, an outcome not achieved by glycemic control through insulin administration [25]. However, there are some contrary opinions [26]. Tight glucose control benefits parenteral nourishment in critically ill patients receiving frequent, accurate glucose monitoring and a reliable insulin treatment protocol [27, 28]. The stress hyperglycemia ratio (SHR), defined as the ratio of mmol/L blood glucose and % hemoglobin A1c (HbA1c), has been used to predict the risk of rehospitalization for chest pain [29]. Elevated blood glucose levels at the time of admission have been linked to negative clinical results in individuals with cardiovascular diseases. A U-shaped relationship between the stress hyperglycemia ratio and short-term mortality has been observed in patients in the cardiac intensive care unit [30, 31]. HG has been shown to adversely affect clinical outcomes, leading to higher mortality and morbidity. The efficacy and safety of blood glucose control without early parenteral nutrition is unclear [32]. Leuven randomized controlled trials (RCTs) suggested that intermediate glucose control was worse than strict glucose control. Leuven, along with the NICE-SUGAR study, suggested that the intermediate blood glucose index (<10 mmol/L) was the optimal level [33]. In addition, several previous investigations found that the optimal range of blood glucose levels was 5.27–6.94 mmol/L when the risk ratios of all-cause deaths were lowest. The consensus of experts on the glycemic management of critically ill patients suggests maintaining blood glucose levels at 6.1–7.8 mmol/L in non-diabetic critically ill patients (Grade 2+, weak recommendation) [34, 35, 36, 37]. Our research considers the optimal level of the initial blood glucose for critically ill non-diabetes patients with HF to be around 7 mmol/L.

Our study has several limitations that warrant careful consideration. First, the cross-sectional nature of our research design poses challenges in inferring causation from the observed associations between initial blood glucose levels and hospital mortality. This design restricts our understanding of the temporal sequence of events, which is essential for establishing a cause–effect relationship. Second, we underscore the need for caution when extrapolating our findings to other populations not represented in the MIMIC database. We also suggest that future research should seek to validate our findings using data from different healthcare systems and populations to ensure the robustness and applicability of the results. Third, although we have endeavored to account for numerous confounding variables, our analysis did not include factors such as historical glycemic control, glycosylated hemoglobin levels due to the unavailability of this data and the classification of heart failure, disease progression, treatment modalities, complications, patient’s physical condition, and genetic factors in contributing to patient outcomes. These variables could provide additional insights into the complex relationships and interactions affecting mortality rates. The omission of these variables means we cannot fully elucidate the relationship between prior glycemic control and baseline glucose levels. The direction and magnitude of this potential bias are challenging to quantify but must be acknowledged when interpreting the findings in this study.

## 5. Conclusions

The correlation between initial blood glucose and in-hospital mortality in critically ill non-diabetic patients with heart failure is U-shaped. The optimal initial blood glucose level for this group of patients was around 7 mmol/L.

## Data Availability

The raw data supporting the conclusions of this article will be made available by the authors without undue reservation.

## Abbreviations

HF, heart failure; MIMIC-III, Medical Information Mart for Intensive Care-III; RBC, red blood cells; ${\mathrm{SPO}}_{2}$, pulse oxygen; COPD, chronic obstructive pulmonary disease; CHD with no MI, cardiovascular heart disease with no myocardial infarction; eGFR, estimated glomerular filtration rate.
